# Diagnostic value of Chest CT and Initial Real-Time RT-PCR in COVID-19 Infection

**DOI:** 10.12669/pjms.37.1.2956

**Published:** 2021

**Authors:** Ugur Kostakoglu, Aydin Kant, Serhat Atalar, Baris Ertunc, Sukru Erensoy, Enes Dalmanoglu, Ismail Yilmaz, Belgin Sevimli, Ayse Erturk, Gurdal Yilmaz

**Affiliations:** 1 Ugur Kostakoglu, Department of Infectious Diseases and Clinical Microbiology, Faculty of Medicine, Recep Tayyip Erdogan University, Rize, Turkey; 2 Aydin Kant, Chest Diseases Department, Trabzon Vakfikebir State Hospital, Trabzon, Turkey; 3 Serhat Atalar, Department of Infection Diseases and Clinical Microbiology, Niksar State Hospital, Tokat, Turkey; 4 Baris Ertunc, Department of Infection Diseases and Clinical Microbiology, Akcaabat HackalIbaba State Hospital, Trabzon, Turkey; 5 Sukru Erensoy, Department of Infection Diseases and Clinical Microbiology, Yavuz Selim Bone Diseases and Rehabilitation Hospital, Trabzon, Turkey; 6 Enes Dalmanoglu, Department of Infectious Diseases and Clinical Microbiology, Faculty of Medicine, Recep Tayyip Erdogan University, Rize, Turkey; 7 Ismail Yilmaz, Chest Diseases Department, Akcaabat HackalIbaba State Hospital, Trabzon, Turkey; 8 Belgin Sevimli, Department of Ophthalmology, Trabzon Vakfikebir State Hospital, Trabzon, Turkey; 9 Ayse Erturk, Department of Infectious Diseases and Clinical Microbiology, Faculty of Medicine, Recep Tayyip Erdogan University, Rize, Turkey; 10 Gurdal Yilmaz Department of Infectious Diseases and Clinical Microbiology, Faculty of Medicine, Karadeniz Technical University, Trabzon, Turkey

**Keywords:** COVID-19, rtRT-PCR, Chest CT

## Abstract

**Objectives:**

To evaluate the diagnostic value of the rtRT-PCR test and CT in patients presenting with typical clinical symptoms of COVID-19.

**Methods:**

The study with the participation of four center in Turkey was performed retrospectively from 20 March-15 April 2020 in 203 patients confirmed for COVID-19. The initial rtRT-PCR test was positive in 142 (70.0%) of the patients (Group-I) and negative in 61 patients (Group-II).

**Results:**

The mean age of the patients in Group-I was 49.7±18.0 years and the time between the onset of symptoms and admission to the hospital was 3.6±2.0 days; whereas the same values for the patients in Group-II were 58.1±19.9 and 5.3±4.2, respectively (p=0.004; p=0.026). Initial rtRT-PCR was found positive with 83.5% sensitivity and 74.1% PPV in patients with symptom duration of less than five days. It was found that rtRT-PCR positivity correlated negatively with the presence of CT findings, age, comorbidity, shortness of breath, and symptom duration, while rtRT-PCR positivity correlated positively with headache. Presence of CT findings was positively correlated with age, comorbidity, shortness of breath, fever, and the symptom duration.

**Conclusions:**

It should be noted that a negative result in the rtRT-PCR test does not rule out the possibility of COVID-19 diagnosis in patients whose symptom duration is longer than five days, who are elderly with comorbidities and in particular who present with fever and shortness of breath. In these patients, typical CT findings are diagnostic for COVID-19. A normal chest CT is no reason to loosen up measures of isolation in patients with newly beginning symptoms until the results are obtained from the PCR test.

## INTRODUCTION

The coronavirus 2 (SARS CoV-2) pandemic is a global public health problem which cannot be treated until a specific antiviral drug is developed, which renders early detection and medical isolation extremely important. In the diagnosis of COVID-19, reverse transcriptase polymerase chain reaction (RT-PCR) test on respiratory samples is accepted as the gold standard.^1^-^3^ However, the sensitivity is reported as low as 59-71%, because of possible false negative with the RT-PCR test due to insufficient viral material in the sample or a procedural error.^4^,^5^

The time consuming nature of the test and limited supply of the test kit are other problems. Early recognition is crucial to quickly initiate treatment, isolation, and care, not to mention to reduce transmission, including infections in close contacts and healthcare professionals. So far computed tomography (CT) was used as an alternative diagnostic method for detection of cases.^6^,^7^ The aim of this study was to explore the diagnostic value of rtRT-PCR test and CT in patients presenting with typical clinical symptoms of COVID-19.

## METHODS

Medical records of 203 cases treated between 20 March-15 April 2020 in four centers were retrospectively evaluated. The study protocol was approved by the Recep Tayyip University local ethical committee (Protocol number: 05.06.2020 / 40465587-050.01.04-108/2020/81). In the study, patients who were admitted with the typical clinical symptoms of COVID-19 were given an rtRT-PCR test and CT imaging on the first day. For those with a negative rtRT-PCR result, the test was repeated after 24 hours. An antibody test was performed if there was still clinical suspicion despite two negative test results. Patients who were admitted with typical clinical symptoms of COVID-19 and whose rtRT-PCR and/or antibody test returned positive, were evaluated retrospectively. All patients should have initial thorax CT. Patients whose first PCR tests were positive were designated as Group-I, whereas those with the first PCR tests negative, but later PCR tests or antibody tests positive were Group-II. Demographic, clinical, and laboratory data of the patients were recorded in the study form following a review of their files.

### Statistical Analysis

Descriptive statistical analysis was performed for all studied variables. Compatibility with normal distribution of data obtained by measurement was assessed using the Kolmogorov Smirnov test. Student’s t test was used to analyze normally distributed data, and the Mann-Whitney U test was used for non-normally distributed data. The chi square test was used to compare categoric variables. Data obtained by measurement are expressed as mean ± standard deviation. Data obtained by counting are expressed as numbers (%); analyses were performed using the chi-square test. Correlation analysis was performed using Pearson’s correlation test or Spearman’s correlation test. Receiver operating characteristic (ROC) analysis was performed to calculate the sensitivity, specificity, and negative predictive value (NPV) and positive predictive value (PPV) of statistically significant variables. P<0.05 was considered statistically significant.

## RESULTS

A total of 203 patients with COVID-19 diagnosis were included in the study. The mean age of the patients was 52.3±18.9 (9-90) and 121 (59.6%) of them were male and 82 (40.4%) were female. Among these 107 (53.0%) had a comorbidity. The most common comorbidity was hypertension (n=63). There were 142 patients in Group-I and 61 patients in Group-II. In 32 (15.8%) of the patients whose first rtRT-PCR test was negative, the rtRT-PCR test returned positive results on the second day. In 29 (14.3%), only the antibody test was positive. CT findings were consistent with Covid-19 in 87 (61.3%) of the patients in Group-I and 56 (91.8%) in Group-II (P<0.001). The mean age of the patients in Group-I was 49.7±18.0 years and the time between the onset of symptoms and admission to the hospital was 3.6±2.0 days; whereas the same values for the patients in Group-II were 58.1±19.9 and 5.3±4.2, respectively (p=0.004; p=0.026). Of these patients, 48.8% had fever, 61% were coughing, 26.6% had a sore throat, 27.1% shortness of breath, 24.6% myalgia, 42.4% weakness-malaise, 23.2% headache, and 6.4% had complaints of diarrhea. In Group-I, 32 patients (22.5%) complained of shortness of breath and 39 (27.5%) of headache, whereas in Group-II these complaints were seen in 23 (37.7%) and 8 (13.1%) patients, respectively. (p=0.040, OR 0.48; p=0.041, OR=2.51). Table-1 shows the demographic and clinical characteristics of the patients.

**Table-I:** Results of the ROC analysis performed to determine the diagnostic efficacy of rtRT-PCR and CT.

	Parameters	Cut off	AUC	AUC 95%CI	Sensitivity	Specificity	PPV	NPV	P
rtRT-PCR	CT	1	0.653	0.583-0.718	38.7	91.8	91.7	39.2	<0.001
	Age	≤ 60	0.623	0.552-0.690	69.7	47.5	75.6	40.3	0.006
	Symptom duration	≤ 5	0.602	0.527-0.673	83.5	33.9	74.1	47.5	0.027
	Dyspnea	0	0.576	0.505-0.645	77.5	37.7	74.3	41.8	0.035
	Headache	1	0.572	0.501-0.641	27.5	86.9	83.0	34.0	0.013
CT	Age	>44	0.753	0.688-0.811	74.8	65.0	83.6	52.0	<0.001
	Symptom duration	>2	0.726	0.655-0.789	74.5	58.7	84.3	43.5	<0.001
	Dyspnea	1	0.598	0.527-0.666	32.9	86.7	85.5	35.1	0.001
	Fever	1	0.598	0.527-0.666	54.6	65.0	78.8	37.5	0.009

Presence of CT findings were negatively correlated with PCR positivity (r=-0.307; p<0.001), age (r=-0.203; p=0.005), comorbidity (r=-0.243; p<0.001), shortness of breath (r=-0.156; p=0.026), symptom duration (r=-0.271; p<0.001) and a positive correlation was found between PCR positivity and headache (r=0.156; p=0.026). The correlation was positive between the presence of CT findings (r=0.401; p<0.001), comorbidity (r=0.343; p<0.001), shortness of breath (r=0.201; p=0.026), fever (r=0.178; p=0.011) and symptom duration (r=0.346; p<0.001).

**Fig.1:**
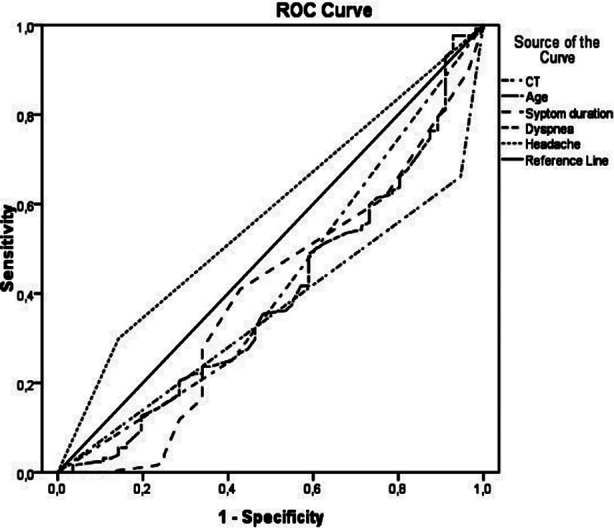
ROC curve between symptom duration and CT findings.

Table-I and Fig.1 show the ROC analysis performed to determine the diagnostic efficacy of rtRT-PCR and CT (Table-I, Fig.1). In the analysis performed, the area under curve (AUC) between the initial rtRT-PCR test positivity and CT was 0.653, the sensitivity was 38.7%, and the specificity was 91.8%. Initial rtRT-PCR was found positive with 83.5% sensitivity and 74.1% PPV in patients with symptom duration of less than five days. In patients under the age 60, rtRT-PCR was found positive with 69.7% sensitivity and 75.6% PPV, in the absence of shortness of breath there was 77.5% sensitivity and 74.3% PPV, and in the presence of headache 86.9% specificity and 83.0% PPV. For patients with symptom duration of less than two days, CT was positive with 74.5% sensitivity and 84.3% PPV. The findings for patients over 44 were 74.8% sensitivity, 83.6% PPV, in the presence of shortness of breath 86.7% specificity and 85.5% PPV, and in the presence of fever 54.6% sensitivity, 65.0% specificity, and 78.8% PPV.

In patients with comorbidities, the PCR positivity was 45.1% and CT positivity was 64.1%. Eighteen (8.9%) patients were hospitalized in the intensive care unit (ICU). All patients in the ICU had comorbidities. Six patients deceased and 153 were released from the hospital. The treatment of another 44 patients continued.

## DISCUSSION

As the world fights the COVID-19 outbreak, reports from China, South Korea, and Singapore show that early detection of the disease and isolation of patients are the most important phase in keeping the spread of the disease under control.^8^ Considering the infectious rate of SARS-CoV-2, it is important to have accurate and precise diagnostic technologies as soon as possible, as false negative test results have a detrimental epidemiological impact against global efforts to contain the epidemic.^9^

Currently, the most convenient and efficient method of COVID-19 scanning is rtRT-PCR testing on samples obtained with throat or nasal swab. However, the reported rtRT-PCR sensitivity for COVID-19 is 50-62%.^10^ It is reported that poor sample quality, too early or delayed sample collection, inconvenient sample storage and transport as well as virus mutations could lead to false negative results.^11^ A number of factors including the viral load in the respiratory system, sample source, sampling procedures and timing, quality control of the test, and the natural performance of test kits can affect accuracy.^10^ While SARS-CoV-2 can most reliably be detected in sputum and then nasal swabs, throat swabs have been reported to be unreliable 8 days after symptom onset.^12^

Our study shows higher rtRT-PCR positivity in patients under 60, with symptom duration of less than five days, presenting with headache and without shortness of breath. In patients under the age 60, rtRT-PCR was found positive with 69.7% sensitivity and 75.6% PPV, in the absence of shortness of breath it was 77.5% sensitivity and 74.3% PPV, and in the presence of headache 86.9% specificity and 83.0% PPV. Shortness of breath showed that the disease descended into the lungs and pointed to a possibility of 52% negativity of the rtRT-PCR test on throat or nasal swab samples (p=0.040, OR=0.48). The presence of headache indicated that the disease was at an initial stage and a 2.51 times greater likelihood of a positive rtRT-PCR test (p=0.041, OR=2.51).

In their study, Guo *et al.*, reported that the PCR positivity rate after the onset of symptoms was higher than 90% on days one to three, lower than 80% on the 6th day, and lower than 50% after 14 days.^13^ In this study, the PCR detection rate was higher than in IgM ELISA before 5.5 days passed after the onset of symptoms; however, the positivity rate of IgM ELISA became higher than PCR after 5.5 days.^13^ This result was consistent with the results of our study. In our study, there was negative correlation between rtRT-PCR positivity and presence of CT findings, age, comorbidity, shortness of breath, and symptom duration and a positive correlation between rtRT-PCR positivity and headache. However, in patients without these characteristics, the sensitivity, specificity, PPV and NPV values of rtRT-PCR were low. For these reasons, rtRT-PCR cannot be accepted as a reliable and independent tool for COVID-19 scanning. Considering that false negatives play an important role in the spread of the infection, alternative diagnostic methods are needed. Furthermore, PCR test kits may not be readily available in many countries or there may not be a sufficient number of staff to perform the test. On the other hand, easily accessible chest CT played an important part in the early detection and evaluation of COVID-19 infection, as well as in monitoring the treatment response at the beginning of the pandemic.^14^ One study noted that in COVID-19 pneumonia, signs of severe lung disease developed on CT about 10 days after the onset of symptoms, and signs of chest CT improvement began about 14 days after the first symptoms began.^15^

In our study, for patients with a symptom duration of less than two days, CT was positive with 74.5% sensitivity and 84.3% PPV. The findings for patients over 44 were 74.8% sensitivity, 83.6% PPV, in the presence of shortness of breath 86.7% specificity and 85.5% PPV, and in the presence of fever 54.6% sensitivity, 65.0% specificity, and 78.8% PPV. Presence of CT findings was positively correlated with age, comorbidity, shortness of breath, fever, and symptom duration. In the literature there are reports that CT can be used as the first research tool in patients presenting a high clinical suspicion for COVID-19.^16^ Other publications also indicate that the frequency of CT findings is related to symptom duration.^17^,^18^ Bernheim *et al*. showed that the CT finding was not sufficient for diagnosis in the first two days of illness, but it acquired diagnostic value in the following days.^18^

### Limitations of the study

The small number of patients included in the study can be seen as the most important limitation. False negativity of the rtRT-PCR test may be another limitation of the study.

## CONCLUSION

Consequently, it is found that the rtRT-PCR test is correlated negatively with patient age, symptom duration, presence of comorbidity, and shortness of breath. In the presence of headache, however, the positivity rate of rtRT-PCR tests increases. Nonetheless, CT is found to be positively correlated with patient age, symptom duration, comorbidity, shortness of breath, and fever. It should be noted that a negative result in the rtRT-PCR test does not rule out the possibility of COVID-19 diagnosis in patients whose symptom duration is longer than five days, who are elderly with comorbidities and in particular who present with fever and shortness of breath. In these patients, typical CT findings are diagnostic for COVID-19. In cases where the rtRT-PCR test cannot be performed or the first rtRT-PCR test is negative, CT imaging bears crucial importance in terms of public health to prevent the spread of infection. In patients with emerging symptoms, a normal chest CT does not warrant loosening isolation measures before the PCR test results are obtained.

### Authors Contribution:

**UK, GY:** Conceived, designed and did statistical analysis & editing of manuscript. They also take the responsibility for integrity of research.

**UK, AK, SA, BE, SU, ED, IY, BS:** Did data collection and manuscript writing.

**UK, AE, GY:** Did review and final approval of manuscript.
